# Narrative approach to understanding compassion: a mixed methods study in a Polish sample

**DOI:** 10.3389/fpsyg.2025.1476446

**Published:** 2025-02-03

**Authors:** Mariusz Zieba, Mateusz P. Zatorski, Natalia E. Wójcik

**Affiliations:** Institute of Psychology, SWPS University, Poznań, Poland

**Keywords:** compassion, narrative psychology, communion, agency, stressful events, mixed methods

## Abstract

**Objectives:**

This study investigates the relationship between compassion, defined by Gilbert as “a sensitivity to suffering in self and others, with a commitment to try to alleviate and prevent it” and narrative identity. We explored whether individuals with high and low levels of compassion differ in narrative characteristics such as affective tone, agency, and communion themes, as well as the use of redemption or contamination sequences in stressful life event narratives.

**Methods:**

A mixed-method study was conducted with Polish adult participants (*N* = 63), half of whom had low and the other half high levels of compassion. Participants completed several questionnaires, including the Compassion Action and Engagement Scale. A few weeks later, they participated in individual interviews where they narrated several key life events. The interviews were recorded, transcribed, and coded by an individual blind to the participants' compassion levels and other identifying information. The self-narratives were analyzed for affective tone, agency and communion themes, and redemption and contamination sequences. Differences in these elements between individuals with low and high compassion were analyzed using *t*-tests.

**Results:**

Our findings indicate that individuals with higher compassion more frequently incorporate themes of agency and communion in their narratives, particularly in stories of failure and the past year's most difficult event. The narrative identity of a highly compassionate person includes more content related to seeing oneself as sensitive to suffering and actively working to reduce or prevent it.

**Conclusions:**

This study highlights how individuals with varying levels of compassion construct narratives about significant life events. Narrative approaches can foster compassionate engagements and actions, potentially improving therapeutic practices and personal development strategies. The results underscore the importance of narrative analysis in understanding compassion and suggest that compassion levels may influence how individuals interpret and narrate their life experiences, offering valuable insights for future research.

## 1 Introduction

Compassion has been a central concept in many philosophical, religious, and psychological frameworks, often emphasized as a fundamental human quality. In Buddhist tradition, compassion is seen as an essential element, characterized by openness to the suffering of others and a commitment to alleviate it (Dalai Lama and Jacobi, 1995). In Christianity, compassion is considered a virtue, encapsulated in the act of “suffering with” others, aligning with the Latin root “compati.” The understanding of compassion as a virtue has roots in ancient Greek philosophy. While the Greeks may not have used the term “compassion” as it is in contemporary discourse, their philosophical explorations laid the groundwork for understanding the importance of empathy, kindness, and rational benevolence in human relationships. Compassion was interwoven with broader ethical principles and the pursuit of a just life, as seen in Plato's works (Ferrari and Griffith, 2000), and a virtuous life, as discussed by Aristotle, who believed that pity involved recognizing that suffering could happen to oneself or one's loved ones, thus invoking a sense of shared humanity and vulnerability (Bartlett and Collins, 2011; McGrath and Brown, 2020).

Contemporary psychology devotes considerable attention to the sources and functional role of compassion. Various definitions of compassion include elements such as sympathy, empathy, noticing and paying attention to distress, generosity, openness, commitment, and courage, among others (Goetz et al., 2010; Strauss et al., 2016).

Some concepts focus on understanding compassion as a specific subjective feeling or emotional reaction to another person's suffering. This aligns compassion closely with sympathy as defined by Davis (1983) or Eisenberg, who characterizes sympathy as “an emotional reaction that is based on the apprehension of another's emotional state or condition and that involves feelings of concern and sorrow for the other person” (Eisenberg et al., 1994, p. 776).

Other definitions also include cognitive and motivational aspects. Goetz et al. (2010, p. 351), based on their systematic review of studies on compassion, define it as: “the feeling that arises in witnessing another's suffering and that motivates a subsequent desire to help.” Similarly, Strauss et al. (2016, pp. 17–18), based on their review of many contemporary definitions of compassion, write that “compassion is seen as awareness of someone's suffering, being moved by it (emotionally and, according to some definitions, cognitively), and acting or feeling motivated to help.”

Lazarus defines compassion as one of the empathic emotions, whose core theme is “being moved by another's suffering and wanting to help” (Lazarus, 1991, p. 289). However, he also notes that “to cope effectively and help those who suffer, we must learn how to distance ourselves emotionally from the emotional significance of their suffering, so it does not overwhelm us” (Lazarus, 1999, p. 246). In this conception, overly intense compassion can trigger avoidance strategies, leading to a reluctance to engage with or help the suffering person. In contrast, the ability and motivation to tolerate distress and uncomfortable feelings that arise in oneself as a result of seeing suffering is a crucial element in Paul Gilbert's (2015) concept of compassion.

Gilbert defines compassion as “a sensitivity to suffering in self and others, with a commitment to try to alleviate and prevent it” (Gilbert, 2017a). In this conceptualization, compassion is a care-focused motivation rooted in evolved caring systems and social mentalities, shaped by humans' advanced cognitive, empathic abilities, and social practices (Gilbert, 2015; Gilbert and Simos, 2022). It involves both recognizing suffering (stimulus detection) and taking actions to address it (response functions), each associated with distinct physiological processes and competencies (Di Bello et al., 2021; Gilbert, 2020; Gilbert and Simos, 2022; Gilbert and Van Gordon, 2023). Recent neuroimaging studies have identified several distinct brain regions associated with compassion, including areas in both the frontal and subcortical regions (Kim et al., 2020; Klimecki et al., 2014; Klimecki and Singer, 2017; Singer and Engert, 2019), highlighting the complexity of the mechanisms underlying this motivation.

Compassion involves two primary processes: motivated attention to suffering (compassionate engagement), with six competencies—motivation to care, sensitivity to distress, empathy, compassion, distress tolerance, and non-judgment; and motivated efforts to alleviate and prevent suffering (compassionate action), encompassing six competencies—helpful attention, imagery, reasoning, sensory focus, feelings and emotions, and behaviors. Importantly, Gilbert argues that compassion must be distinguished from its components. Sensitivity to suffering or needs alone does not necessarily lead to actions aimed at alleviating that suffering or addressing those needs (Poulin, 2017). Moreover, sensitivity to distress without appropriate action may increase vulnerability to depression and anxiety (Gilbert et al., 2017). To be compassionate, individuals must also possess the competencies required to choose appropriate responses, along with the courage and wisdom to undertake actions that counter or reduce suffering, persevere despite obstacles, and achieve intended outcomes (Gilbert and Choden, 2013).

These competencies—encompassing both the skills of compassionate engagement and compassionate action—operate across three flow of compassion: showing compassion to others, receiving compassion from others, and self-compassion (Gilbert et al., 2017). The first orientation corresponds to the understanding of compassion in most contemporary theories, emphasizing the readiness to notice others' suffering and the willingness to help them. Compassion from others involves the willingness to receive help and support from others. In turn, self-compassion entails the readiness to accept one's own suffering and to treat oneself with non-judgmental kindness (Gilbert et al., 2017).

In Gilbert's concept, being compassionate involves possessing competencies for insight, reasoning, mentalizing, and mindfulness (Gilbert, 2017a; Gilbert and Van Gordon, 2023). The development of these competencies is a result of life experiences and the growth of one's identity. As individuals encounter and reflect on various life events and circumstances, they gain deeper insight into their mental states and personal experiences. This process aligns with McAdams' narrative theory, which posits that people construct their identities by integrating life experiences into a cohesive and evolving story (McAdams, 1993, 2001, 2008; McAdams and McLean, 2013). As narrative identity develops, individuals not only gain a better understanding of their psychological states but also become more responsible for their decisions and actions, evolving from mere “actors” to “agents” and eventually “authors” of their life stories (McAdams, 2013),

The process of constructing narrative identity involves storytelling, where individuals recount their life experiences in a meaningful way (McAdams, 2008). People's life stories are not just recounting of events but rather their personal interpretations. Each story can be told from multiple perspectives and in various ways (McLean and Pasupathi, 2012). Constructing narratives about one's life experiences commits adopting the perspectives of both the protagonist and the narrator. The narrator decides which elements to include in the story, what to focus on, and what to omit (Robinson and Hawpe, 1986), even when they are the protagonist of the story. Moreover, life stories can change over time (McAdams, 2008); for example, a painful childhood event once told as a terrible failure and embarrassment may later become a humorous anecdote.

The elements of an event and how they are framed in a personal narrative depend significantly on individual characteristics, including personality traits, and the social context—culture being an important source of prevailing storytelling patterns (Hammack, 2008). On the other hand, understanding an individual's specific way of framing experiences in narrative form can provide valuable insights into what is important to that person and how they think about themselves, the world, and others. For the individual, narrative is a way of understanding themselves and the world (Bruner, 1991), while for the listener or reader, what and how the person tells their story can aid in better understanding them.

A growing body of research provides extensive data on the relationships between various properties of narrative identity and different aspects of human functioning and wellbeing (Adler et al., 2016). Researchers interested in self-narratives may focus on both narrative content and thematic components (Adler et al., 2016). Studies using qualitative methods such as thematic analysis (Braun and Clarke, 2022) or interpretative phenomenological analysis (Smith et al., 2009) can facilitate a deeper understanding of the individual experiences, beliefs, and perspectives of individuals. Other types of research concentrate on identifying differences in specific linguistic and structural properties of narratives, as well as thematic components such as affective tone and motivational themes.

Each person has a characteristic affective tone that permeates their self-narratives, shaped by childhood emotional experiences (McAdams et al., 2006). This tone influences how narratives about different life events are constructed. People with a positive affective tone in their self-narratives tend to expect positive outcomes from their current engagements and seek positive aspects of past events. A positive affective tone is associated with higher life satisfaction and psychological wellbeing (McAdams et al., 2001). Affective tone is considered a relatively stable individual characteristic, remaining consistent over time. McAdams et al. (2006) found that narratives about important events, told by participants 3 years apart, had a similar affective tone, with a correlation of 0.43, even though the events described were usually different from those told initially.

Self-narratives can have a negative, neutral, or positive affective tone, but they can also include specific dynamics: contamination sequences (positive beginning and negative ending or consequences of an event) or redemption sequences (negative beginning and positive ending or consequences). According to McAdams et al. (2001), the redemption sequence explains life satisfaction and psychological wellbeing more strongly and independently of affective tone (McAdams et al., 2001).

Agency and communion, introduced by Bakan (1966), are fundamental dimensions of human behaviors. Agency refers to the capacity for self-assertion, control, and mastery, encompassing traits like independence and self-efficacy. In contrast, communion involves the capacity for forming connections, relationships, and concern for others, highlighting traits such as empathy and cooperation. McAdams (1985; McAdams et al., 1996) asserts that agency and communion are two modalities that appear in all narratives in which people describe their experiences. In narrative themes, communion is characterized by stories that emphasize love, intimacy, and belonging, while agency is characterized by stories that highlight personal achievement, independence, and the ability to influence one's own life and surroundings. The prominence of agency and communion themes varies across individuals' life stories, and these differences can be quantified.

While agency and communion are distinct dimensions, they often correlate in individual narratives. This balance allows individuals to see themselves as both competent and connected, capable of influencing their lives while being part of a larger community. Research suggests that a narrative identity that includes strong themes of both agency and communion is associated with higher levels of life satisfaction and psychological health (McAdams et al., 1996).

Narrative analyses can also focus on more indirect indicators characterizing an individual's ways of experiencing events. Research in psychology has extensively explored the use of pronouns and verbs as indicators of various psychological states, interpersonal dynamics, and social behaviors. Singular and plural forms of these linguistic elements often reflect underlying cognitive and emotional processes (Chung and Pennebaker, 2007; Pennebaker and King, 1999). For example, Pennebaker (2011) found that frequent use of first-person singular pronouns is associated with depression and anxiety, indicating a more inward, self-referential focus often linked to negative affect. Conversely, the use of third-person singular pronouns can indicate social distance or objectivity (Pennebaker et al., 2003). On the other hand, the frequent use of first-person plural pronouns signifies social inclusion and collective identity, often associated with group cohesion, shared experiences, and positive relational outcomes. Moreover, individuals who frequently use plural pronouns have a greater ability to adopt others' perspectives, which is crucial for empathetic interactions (Kacewicz et al., 2014). Individuals who frequently use singular pronouns may be more self-focused and have higher levels of self-awareness but also a higher incidence of depression (Rude et al., 2004). Although no studies have specifically examined the linguistic patterns of individuals with varying levels of compassion, related findings suggest that individuals with higher compassion levels may use more first-person plural verbs and pronouns, reflecting perspective-taking and social inclusion. In contrast, lower compassion levels may be associated with a self-referential focus, marked by frequent use of first-person singular verbs and pronouns and third-person singular pronouns.

To our knowledge, no studies have examined the formal linguistic properties characteristic of individuals with low vs. high levels of compassion. However, based on the reviewed research findings, it can be hypothesized that individuals with higher levels of compassion are relatively more likely to use first-person plural verbs and pronouns. In contrast, the language of individuals with lower levels of compassion may be characterized by a more frequent use of first-person and third-person singular verbs and pronouns in their narratives.

The present study aimed to examine whether differences in compassion levels are associated with specific narrative characteristics. We selected these characteristics from motivational and affective themes that, according to Adler et al. (2017), are among the most frequently analyzed in studies on narrative identity. These include affective tone, the prominence of agency and communion themes, and the use of redemption or contamination sequences. The study was conducted in a cross-sectional design, and we did not intend to determine whether narrative properties influence compassion levels or whether compassion is expressed in the aspects of life stories that narrators focus on and how they present them.

Existing literature suggests that high compassion is associated with an interest in others and a readiness to respond to their suffering (Gilbert, 2017a,b). For individuals inclined to emphasize communion themes in their life event narratives, motivational ideas concerning closeness with others, sharing, nurturance, and caregiving are essential. Thus, we expect a positive relationship between compassion and the theme of communion. Compassion also includes a significant motivational aspect involving readiness to act, engage, and exhibit courage, linking it intricately with agency. Agency, as defined by McAdams (1993) and McAdams et al. (1996), includes personal strength, a need for active engagement, and achievement. However, agency can also involve autonomy or even separation. Therefore, the relationships between agency and compassion may be complex, but a generally positive relationship can be expected.

Analyzing potential links between compassion and emotions requires distinguishing between the long-term consequences of having high compassion and the immediate emotional responses to specific situations. Research has shown that compassion is associated with greater emotional wellbeing and lower levels of negative emotions such as anxiety and depression (Asano et al., 2020; Gilbert et al., 2017). Compassion toward others and from others can also enhance positive emotions such as joy and contentment by promoting altruistic behaviors and social connectedness. However, in distressing situations, including witnessing others' suffering, compassion might increase negative emotions like fear or sadness but simultaneously provides resources to alleviate and tolerate these emotions (Gilbert and Van Gordon, 2023). Additionally, self-compassion can sometimes reduce the intensity of negative self-conscious emotions such as shame and guilt.

The affective tone of narratives, according to McAdams (2001), is shaped in early childhood and will be positive when the child experiences care, safety, and trust. The development of compassion also seems to be linked to what an individual has experienced in life and the beliefs formed about themselves and others, as well as specific competencies developed (Gilbert, 2017a; Gilbert and Van Gordon, 2023). As a result, we can assume that, generally speaking, people whose life stories exhibit a positive affective tone are likely to have high dispositional compassion. This does not necessarily mean that the narratives of people with high compassion about specific, difficult, or stressful life events will also have a more positive affective tone than those of people with low compassion.

The final area of our interest was the potential relationship between compassion and the tendency to use singular vs. plural pronouns and verbs in narratives, in the 1st, 2nd, and 3rd person. Based on previously presented research findings (Pennebaker, 2011; Pennebaker and King, 1999), we expected that the use of first-person and 2nd-person plural pronouns (“we,” “our,” “us,” “they,” “them,” “their”) typical for individuals with a greater tendency to cooperate with others and a sense of closeness would be more frequent among those with high compassion.

We hypothesized that narratives about stressful life events constructed by individuals with higher compassion, compared to those with lower compassion, would:

- have a more positive affective tone;
- more frequently emphasize communion themes;
- moderately more often emphasize agency themes;
- incorporate redemption sequences more frequently (moderate to low relation);
- use first-person singular and plural verbs more frequently in their narratives.

## 2 Materials and methods

### 2.1 Preliminary study and recruitment

To address the research questions, a mixed-method study was conducted with a group consisting of half of the participants having low and the other half having high levels of compassion. Recruitment involved a preliminary phase aimed at identifying individuals with high vs. low levels of compassion.

Participants for the preliminary phase were recruited through advertisements posted on social media in local groups targeting residents of Poznań (a large city in western Poland) and surrounding areas. Participation was voluntary, and participants were entered into a draw to win three shopping vouchers worth ~120 euros each. They were also informed that some of them would be invited to the second phase of the study, which involved interviews about significant life events to be conducted on the Poznań campus of SWPS University. The study procedure was approved by the university's research ethics committee (no. 2021-80-11), and data were collected in accordance with the 1964 Helsinki Declaration.

The preliminary study was conducted online using the Qualtrics platform (https://www.qualtrics.com). Participants completed several questionnaires, including the Compassion Action and Engagement Scale (CEAS-PL), and answered questions about their involvement in various forms of psychological interventions and treatments. Exclusion criteria included current participation in psychiatric treatment, psychotherapy, or compassion training.

The preliminary study group, who completed the full procedure (*N* = 378), consisted of 210 females, 165 males, and three individuals identifying as another gender, aged 18–67 years (*M* = 26.65, *SD* = 7.94). Education levels included 16 with primary and vocational education, 98 with secondary education, 131 pursuing higher education, 129 completed higher education, and the remainder declared “other” forms of education.

### 2.2 Participants

For the second phase of the study, we invited 10% (38 individuals) of the preliminary study participants who scored the lowest and 10% (another 38 individuals) who scored the highest on the CEAS-PL. The CEAS-PL consists of three scales, each measuring a different orientation of compassion (see description below). As such, the selection criterion was the average score across the three scales: Compassion to Self, Compassion to Others, and Compassion from Others.

Not all invited participants decided to take part in this phase. The most common reasons for declining were lack of time or the inability to participate in the study conducted in person at the university. Participants who completed the entire procedure received compensation in the form of a shopping voucher worth ~20 euros. The final number of participants was 63:

#### 2.2.1 Sample 1—low level of compassion

The participants in this group (*n*_1_ = 31) included 15 women and 16 men aged 19–57 years (*M* = 29.60, *SD* = 8.57). Education levels included eight with secondary education, seven pursuing higher education, 14 who completed higher education, and two who declared “other” forms of education. Fourteen study participants declared being in a relationship (seven formal, six informal, and one engaged), and 16 were single.

#### 2.2.2 Sample 2—high level of compassion

This group (*n*_2_ = 32) comprised 19 women and 13 men, with ages ranging from 19 to 46 years (*M* = 25.94, *SD* = 7.14). The educational background of the participants was as follows: six had secondary education, 16 were pursuing higher education, nine had completed higher education, and one reported “other” forms of education. Among the participants, 15 were in informal relationships, 14 were single, and two were divorced.

The average score for the CEAS-PL scales for Sample 1 (low compassion) was 47.64 (*SD* = 7.13), and for Sample 2 (high compassion) it was 75.27 (*SD* = 7.23). The difference between these means was highly strong: *t*_(59)_ = 15.01; *p* < 0.001, Cohen's *d* = 3.84.

### 2.3 Procedure

To explore the differences between participants belonging to groups with low or high levels of compassion, a mixed-method approach was used. Quantitative data from the responses to the questionnaires utilized in the preliminary phase of the study were analyzed. Qualitative data collected during the second phase of the study included the content of narrative interviews conducted with participants a few weeks after they completed the questionnaires.

#### 2.3.1 Questionnaires

**The Compassionate Engagement and Action Scales** (CEAS) comprise three subscales, aligning with Gilbert et al.'s (2017) theory, corresponding to three flows of compassion: Compassion to Self, Compassion to Others, and Compassion from Others. Each subscale is divided into two sections. The first section contains eight statements, each relating to motivation and commitment in coping with discomfort and tension. The second section contains five statements, each relating to ways of using compassion in coping with emotions, thoughts, and situations that cause discomfort and tension. Respondents select answers on a scale of 1–10, indicating how often they act in the specified way (from never to always). The Polish version of the scale (CEAS-PL), was used in the study. The CEAS-PL scales demonstrate good internal consistency, test-retest reliability, and convergent and discriminant validity.

**The Modified Differential Emotions Scale** (mDES) was used to measure positive and negative emotions (Fredrickson, 2013; Fredrickson et al., 2003). Participants indicated their frequency of experiencing each emotion over the past 2 weeks on a five-point scale (0 = not at all, 4 = most of the time) for 10 positive emotions: amusement, awe, contentment, gratitude, hope, inspiration, interest, joy, love, and pride, and nine negative emotions: anger, shame, fear, disgust, embarrassment, guilt, sadness, contempt, and stress. The study used the Polish version translated with permission from the author by Machlah and Zieba (2021). The scale is characterized by high reliability and convergent and discriminant validity.

**Ten Items Personality Inventory** (TIPI) is a brief 10-item scale developed by Gosling et al. (2003) to measure personality traits within the Big Five framework (Costa and McCrae, 2008). The questionnaire consists of 10 items, with two items dedicated to each of the five traits: extraversion, agreeableness, conscientiousness, neuroticism, openness to experience. Each item pair includes one positively-worded and one negatively-worded statement, rated on a seven-point Likert scale ranging from 1 (strongly disagree) to 7 (strongly agree). The study utilized the Polish adaptation of the TIPI by Łaguna et al. (2014). This adaptation has been shown to maintain the reliability and validity of the original English version.

#### 2.3.2 Narrative data collection

Participants from the preliminary phase of the study who scored the lowest or highest on the CEAS-PL were invited by phone to participate in the second part of the study, which involved a narrative interview. They were given the option to choose a convenient time for the interview. The interviews were conducted in a laboratory at the SWPS University building in Poznań, in a soundproof room equipped with audio recording devices. With the written consent of the interviewees, the interviews were recorded and subsequently transcribed manually by a human transcriber. This process ensured that all spoken words were documented, and elements such as pauses in speech and emphasis on certain words were marked.

Each interview was conducted individually by one of two master's psychology students who had undergone training in conducting narrative interviews. The interviewers were informed that one of the study's goals was to explore differences between participants with varying levels of compassion; however, they were not told which group (low or high compassion) the participants belonged to, nor were they aware of the questionnaire results.

The procedure for the interviews was based on the Life Story Interview (McAdams, 2007, 2008). In our study, we used the parts of the Life Story Interview related to life chapters and life challenges. The interviewees were asked to describe nine selected life events: high point, low point, turning point, positive childhood memory, negative childhood memory, life challenge, health challenge, interpersonal loss, failure (regret), and a significant event from last year. For the analysis, we selected narratives from four categories that most frequently included content related to stressful life events. Below are the instructions for the selected categories. The first three were taken directly from the Life Story Interview procedure (McAdams, 2007), while the fourth was developed by us specifically for this study based on that procedure:

**Turning point**. In looking back over your life, it may be possible to identify certain key moments that stand out as turning points—episodes that marked an important change in you or your life story. Please identify a particular episode in your life story that you now see as a turning point in your life. If you cannot identify a key turning point that stands out clearly, please describe some event in your life wherein you went through an important change of some kind. Again, for this event please describe what happened, where and when, who was involved, and what you were thinking and feeling. Also, please say a word or two about what you think this event says about you as a person or about your life (McAdams, 2007, p. 4).
**Life challenge**. Looking back over your entire life, please identify and describe what you now consider to be the greatest single challenge you have faced in your life. What is or was the challenge or problem? How did the challenge or problem develop? How did you address or deal with this challenge or problem? What is the significance of this challenge or problem in your own life story? (McAdams, 2007, p. 4).
**Failure, regret**. Everybody experiences failure and regrets in life, even for the happiest and luckiest lives. Looking back over your entire life, please identify and describe the greatest failure or regret you have experienced. The failure or regret can occur in any area of your life—work, family, friendships, or any other area. Please describe the failure or regret and the way in which the failure or regret came to be. How have you coped with this failure or regret? What effect has this failure or regret had on you and your life story? (McAdams, 2007, p. 4).
**Major event from last year**. Finally, I would like to ask about the most challenging situation you experienced in the past 12 months. This could have been an event involving a serious threat to your health or life, or another type of event that was particularly difficult for you and associated with intense negative emotions. Please describe this event in detail. Tell me how the event came about, where it happened, who was involved, what you were doing, feeling, and thinking during the event, what happened next, and how it ended. Please narrate the event as thoroughly and accurately as possible, providing all relevant facts and circumstances—step by step, what happened.

The instructions provided to the interviewers did not include any references to compassion to avoid suggesting this topic to the participants. The role of the interviewer, in accordance with the Life Story Interview methodology, was limited to listening to what the participants shared and showing interest. Commenting on the participants' stories, giving advice, or probing further was excluded—except for encouraging the participants to elaborate with questions like “Is there anything else you would like to add?” or “Was there anything else important to you in this story?”

The duration of the interviews ranged from ~22 to 90 min (*M* = 48:26, *SD* = 17:37) in Sample 1 (low compassion) and from 15 to 91 min (*M* = 47:34, *SD* = 19:14) in Sample 2 (high compassion). The average duration of the interviews in both groups did not differ significantly: *t*_(61)_ = 0.19, *p* = 0.85, Cohen's *d* = 0.05. The word count ranged from 3,700 to 14,207 (*M* = 7200.87, *SD* = 2808.43) in Sample 1, and from 2,704 to 14,573 (*M* = 7406.97, *SD* = 3011.48) in Sample 2. The word count differed slightly: *t*_(61)_ = 2.01, *p* = 0.049, Cohen's *d* = 0.51.

### 2.4 Analysis

Statistical analyses of the study's quantitative data were performed using IBM SPSS Statistics for Windows, Version 29.0 (IBM Corporation, 2022).

Qualitative data from the narrative interviews were coded for the presence of agency and communion themes, affective tone, and the use of emotional sequences of redemption and contamination. As a result of this coding procedure, each analyzed scene was rated on Likert scales for the intensity of these specific characteristics. This allowed for the comparison of the significance of mean differences between the samples (low vs. high compassion). We also used computerized text analysis to identify formal language properties, such as the use of singular or plural pronouns and verbs. Subsequently, we used statistical tests to check for differences between the groups in terms of the grammatical forms used in the narratives.

The coding was conducted by the second author (MPZ), a psychologist specializing in clinical psychology with over a decade of experience in both research and clinical practice. He is also an expert in the field of compassion and a co-author of several scientific articles on the topic. MPZ had prior experience in analyzing narrative interview data. Additionally, before coding the data from this study, he was trained in the definitions of each coded category and the coding instructions. The coder training followed a three-step procedure: providing and discussing a detailed coding manual, practicing with sample data from the dataset, and revising the manual based on initial questions and discussions to ensure clarity and consistency (Adler et al., 2017). MPZ was aware of the study hypotheses but was blind to all identifying information on the participants.

The coding manual included definitions and detailed descriptions of the constructs: agency and communion (McAdams et al., 1996), affective tone (McAdams et al., 2001), and redemption and contamination sequences (McAdams et al., 2001), prepared based on literature, as well as detailed coding instructions:

**Affective tone** refers to the overall negativity or positivity of a narrated story or writing style (McLean and Breen, 2009). It is the narrative that is evaluated, not the event being described. For instance, an objectively sad event (e.g., being rejected for a date) may be told lightly and humorously. Coders rated the narrative on a five-point Likert scale. If the narrator devotes much attention to describing the negative emotions and feelings they experienced during the event, the affective tone should be rated as 1—strongly negative. Conversely, if the narrative focuses extensively on positive emotions and feelings, the affective tone should be rated 5—strongly positive. Intermediate scale points should correspond to varying levels of negativity/positivity in the affective tone, with a score of 3 indicating an ambivalent tone—where negative and positive emotions are included in the description.

**Redemption and Contamination Sequences**—Narratives about specific life events can differ in their overall affective tone. However, they can also be constructed based on two emotional sequences: Redemption or Contamination (McAdams et al., 2001). In a narrative based on the Redemption sequence, the narrator describes an event in which a story that starts negatively leads to a positive outcome—evil is redeemed or turns out to lead to some good. In contrast, a narrative constructed around the Contamination sequence begins positively but unfolds so that the ending is negative—initial goodness is destroyed, or its outcomes are unfavorable or harmful. Narratives were coded for redemption and contamination using a coding scheme that indicates, for each episode, whether redemption and contamination are present (1) or absent (0).

**Themes of Agency and Communion**—The intensity of the motivational theme of agency is reflected in narratives where the protagonist initiates actions, adopts a task-oriented approach to problems, and seeks to satisfy needs for achievement and respect. In contrast, the theme of communion is evident in narratives emphasizing closeness with others, the value of relationships, willingness to help, and a focus on fulfilling the need for affiliation. Coders were trained using McAdams's (2001a) guidelines, which provide detailed descriptions and examples of subthemes: self-mastery, status, achievement/responsibility, and empowerment for agency; and love/friendship/dialogue, care/help, and community for communion. In this study, coders assessed agency and communion, in general, using a five-point Likert scale, where one indicated the theme was not evident in the narrative content, and five indicated the narrative was dominated by themes in which the protagonist defines themselves as a person with a high level of agency or communion, respectively.

To illustrate the coding system, Table 1 presents examples from participants' narratives. In the first example, high agency (5) and low communion (1) are evident, as the narrator emphasizes self-reliance and problem-solving: “I noticed that after [crying], I have more resources to keep going and do what I need to do.” The narrative lacks focus on interpersonal connections. The second example reflects low agency (1) and high communion (4), centering on mutual support and emotional closeness: “We're always there for each other… I can call in the middle of the night because my oven broke, or just because I'm feeling sad.” Here, relationships take precedence over individual control. The third example demonstrates both high agency (5) and high communion (4), as the narrator describes a transformative experience: “It really gave me this impulse to think about what I could do with my life […] Now, I work especially with people with disabilities, and I think it's a bullseye.” This narrative blends personal agency with a strong commitment to helping others, exemplifying both themes.

**Table 1:** Quotations from participants illustrating coding systems.

**Score on coding system of agency**	**Score on coding system of communion**	**Quotation from participant**
5	1	“Recently, I had a bit of financial trouble, and my first reaction was, you know, very emotional […] maybe you need to let yourself have those thoughts and that stress, I don't know, maybe even crying, because I cried a lot back then. But I noticed that after that, I have more resources to keep going and do what I need to do. So, I kept going […] I mean, I'm very task-oriented, I always have been, but now I've combined it with giving myself space for emotions. Like, I can experience those emotions, and no, I don't see them as a sign of weakness, but I also know that later, something needs to be done. Because […] I'll have my emotions, I'll spend time with them, but then I'll also have the strength to do something. And that's how I dealt with that crisis. Now, I earn enough to cover the basics, but I can also afford a bit more […]”
1	4	“I found out that my best friend is having serious financial difficulties, including debts, like unpaid loans he never wanted to admit to […] That was hard for me […] but we knew that the most important thing now was to support him. […] Now we're so sure that we're always there for each other, that we can meet up, that, I don't know, I can call in the middle of the night because my oven broke, or just because I'm feeling sad, and we know we'll meet up, that it's an option […].”
5	4	“Being alongside someone dealing with such a complex disability for those 3 weeks was, well, it was a brutal, brutal confrontation […]. It really gave me this impulse to think about what I could do with my life […] I changed my studies to something that gives me the opportunity to work, especially with people with disabilities, and I think it's a bullseye […] Back then, I had different ambitions, and I didn't really realize my actual, maybe not abilities, but kind of predispositions, this knack for working with others, especially with people who need help. And truly helping, and seeing that there are results, seeing progress, seeing happiness in these people—it really makes me feel, I don't know, useful, that what I'm doing actually matters to them. And knowing that we're contributing to something happening, to giving them a sense of happiness—it's very rewarding.”

To verify the consistency of coding with the coding manual, another member of the research team (NW, the third author) served as the reliability coder. NW is a psychologist with extensive experience in coding narrative interview data. Using the same coding manual, she evaluated the narratives from a randomly selected sample of 21 participants, which constitutes ~30% of the data. Although Adler et al. (2017) and other experts (Lilgendahl and McAdams, 2011; McLean and Pratt, 2006) suggest coding around 20% for reliability, we chose to compare ratings for a larger portion of the sample due to the relatively high number of coded categories, which could pose potential difficulties in coding.

For the narrative properties coded in the study on a Likert scale (agency, communion, affective tone), we used the ICC coefficient (Shrout and Fleiss, 1979) to assess inter-rater reliability. ICC estimates and their 95% confidence intervals were calculated using SPSS based on a mean-rating (*k* = 2), absolute-agreement, two-way random model. The results were as follows: ICC for agency = 0.820 (95% CI: 0.722, 0.883), ICC for communion = 0.679 (95% CI: 0.506, 0.791), and ICC for affective tone = 0.764 (95% CI: 0.638, 0.847). According to Koo and Li (2016), ICC values between 0.50 and 0.75 indicate moderate reliability, and values >0.75 indicate good reliability. Therefore, our results indicate moderate (communion) to good (agency and affective tone) inter-rater reliability.

Narrative construction based on redemption and contamination sequences was measured on a nominal scale (yes/no). The distributions for both variables were highly skewed, as the vast majority of narratives were rated by both coders as not containing any sequences. In this situation, following Adler et al.'s (2017) recommendation, we used the Delta index (Andrés and Marzo, 2004) and calculated it using the DeltaMAN package (Maldonado et al., 2022). The results obtained for contamination were Delta (overall) = 0.788, and for redemption, Delta (overall) = 0.518. These values indicate a high level of agreement between coders for contamination sequences and a moderate level of agreement for redemption sequences.

We also calculated the frequency of specific grammatical forms in the narratives, such as singular and plural pronouns and verbs in the first, second, and third person. For this purpose, we used the Literary Exploration Machine program (Maryl et al., 2018), which is a web-based application developed within the CLARIN research infrastructure (Lenardič and Fišer, 2022). It is designed for textual scholars to analyze and explore texts in Polish.

## 3 Results

Analyses were conducted to examine differences between individuals with low and high levels of compassion in personality traits and the frequency of experiencing positive and negative emotions. Table 2 summarizes the results of these analyses, which were performed using independent samples *t*-tests, with effect sizes calculated using Cohen's *d*.

**Table 2:** Differences between high and low compassion groups in personality traits and positive and negative emotions.

	**Low compassion**		**High compassion**		***t*_(59)_**	***p***	**Cohen's *d***
**Measures**	***M***	***SD***	***M***	***SD***			
**TIPI**							
Openness to experience	8.80	3.10	10.29	2.08	2.21	0.032	0.57
Conscientiousness	9.53	3.18	10.00	3.14	0.57	0.586	0.14
Extraversion	8.03	4.00	9.97	2.93	2.16	0.036	0.55
Agreeableness	9.77	3.02	10.90	2.84	1.51	0.136	0.39
Neuroticism (inverse)	9.17	3.18	9.32	3.13	0.19	0.848	0.04
**mDES**							
Positive emotions	21.52	7.06	22.97	7.34	0.75	0.456	0.20
Negative emotions	24.18	8.09	24.17	7.52	0.06	0.996	0.02
Love	2.48	1.25	3.24	1.09	2.43	0.010	0.65
Shame	2.67	1.21	2.10	1.20	1.75	0.043	0.47

Cohen's *d* is a standardized measure of the difference between two means, calculated as the difference between the group means divided by the pooled standard deviation. According to Cohen (1988), effect sizes of 0.2 are considered small, 0.5 moderate, and 0.8 large, providing a standardized method to interpret the magnitude of differences, independent of sample size.

The results show significant differences in two personality traits: Openness to Experience and Extraversion, both with medium effect sizes. These findings suggest that individuals with high levels of compassion tend to score higher on these traits compared to those with lower levels of compassion. No significant differences were observed for the remaining traits (Conscientiousness, Agreeableness, and Neuroticism), and the corresponding effect sizes were small.

Regarding positive and negative emotions, there were no significant group differences in the overall frequency of positive or negative emotions. However, significant differences were identified for two specific emotions. Participants with higher levels of compassion reported experiencing the positive emotion of love (“I felt love, closeness, trust”) more frequently, with a large effect size. Conversely, participants with lower levels of compassion experienced the negative emotion of shame (“I felt ashamed, humiliated, disgraced”) more frequently, with a moderate effect size.

In the next step, we analyzed the differences between groups in narrative characteristics such as affective tone, the intensity of communion and agency themes, and redemption and contamination sequences separately for each category of life event.

No significant differences were found between the groups in terms of affective tone or redemption and contamination sequences for any type of life event. This suggests that individuals with low and high levels of compassion do not differ in the affective tone of their narrated important life events, similar to how they do not differ in the frequency of experiencing various emotions over the past 2 weeks as reported in self-descriptive questionnaires.

There were also no significant differences in communion and agency for narratives about turning points and life challenges. However, significant differences were observed in narratives about events related to failure. The average level of communion was significantly higher in the high compassion group (*M* = 1.41, *SD* = 0.88) compared to the low compassion group (*M* = 1.06, *SD* = 0.36): *t*_(61)_ = 2.04; *p* = 0.024, Cohen's *d* = 0.51. Significant differences also emerged for agency themes, which were more frequent in narratives from the high compassion group (*M* = 1.88, *SD* = 1.29) than the low compassion group (*M* = 1.56, *SD* = 1.01): *t*_(61)_ = 1.68; *p* = 0.049, Cohen's *d* = 0.42.

Similar differences were found in narratives about the most challenging event from the past year. The communion theme appeared more frequently in the high compassion group (*M* = 2.25, *SD* = 1.16) than in the low compassion group (*M* = 1.61, *SD* = 0.80): *t*_(61)_ = 2.52; *p* = 0.007, Cohen's *d* = 0.64. The agency theme was also more frequent in the high compassion group (*M* = 1.88, *SD* = 1.29) than the low compassion group (*M* = 1.42, *SD* = 0.81): *t*_(61)_ = 1.69; *p* = 0.049, Cohen's *d* = 42.

The Cohen's *d* values suggest that the differences in communion between the high and low compassion groups for narratives about these two types of life events are moderate, and rather small for agency.

**Figure 1:**
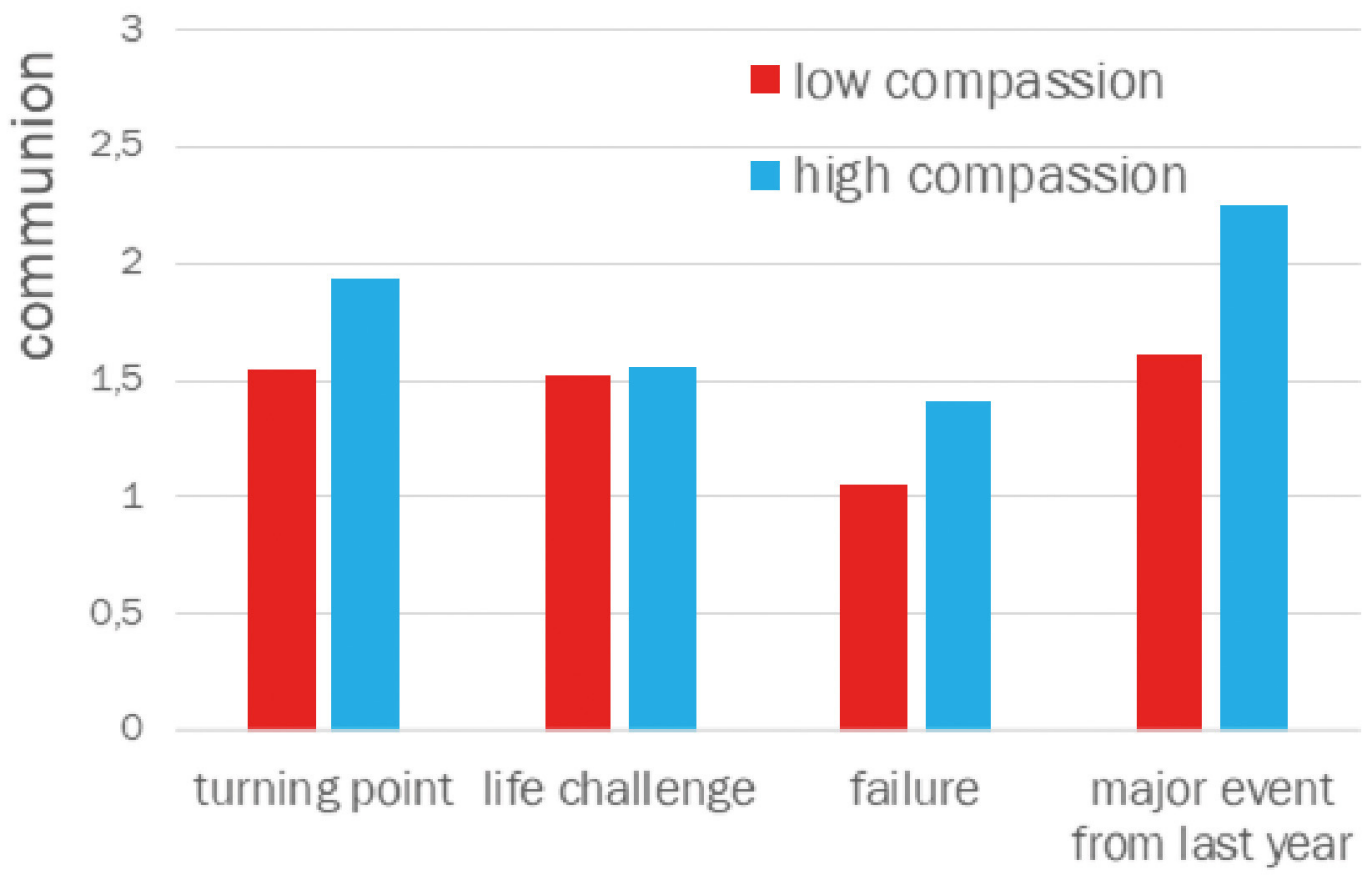
Communication in narratives about turning point, life changes, failure (regret), and major event from last year.

**Figure 2:**
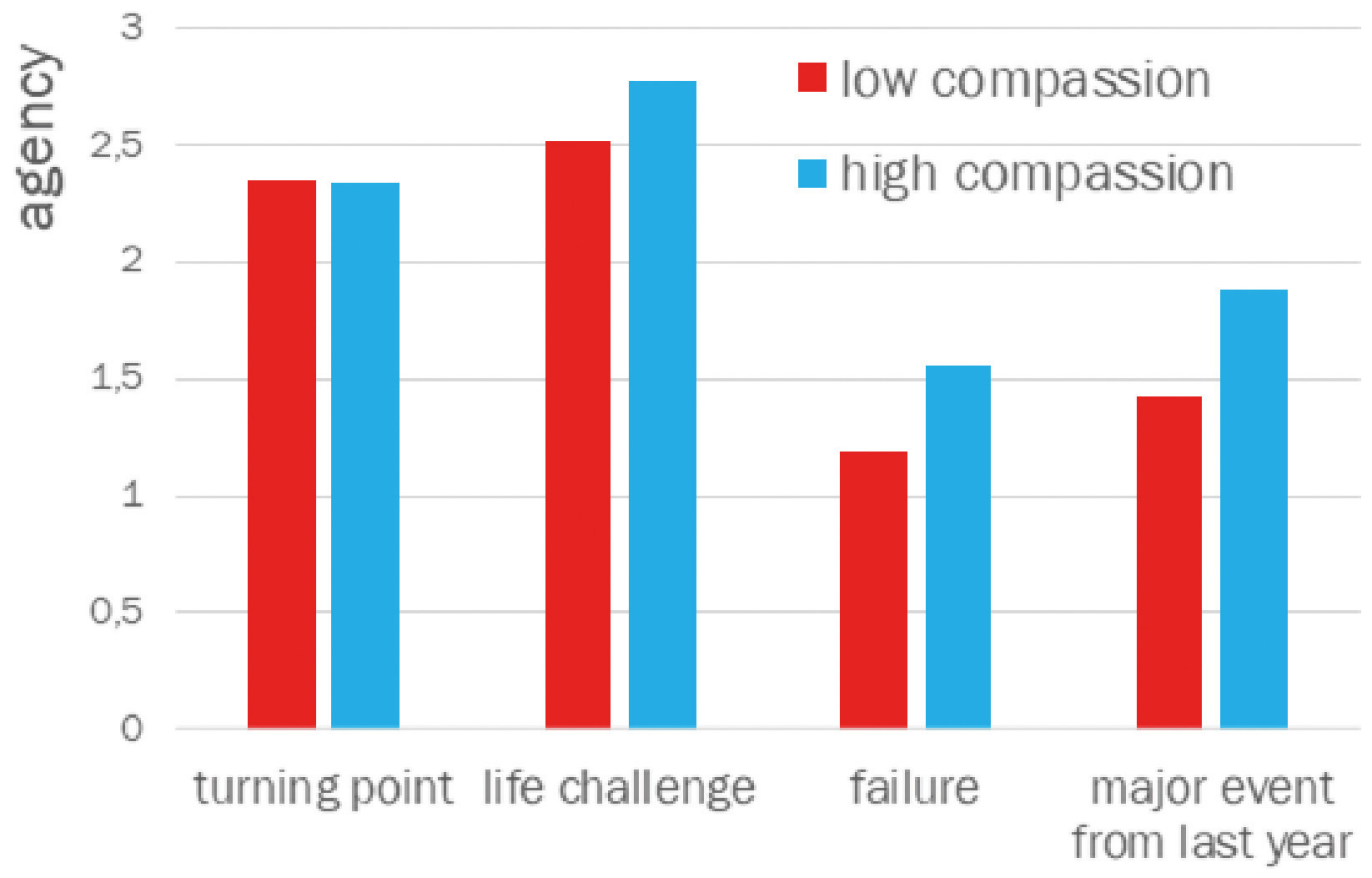
Agency in narratives about turning point, life changes, failure (regret), and major event from last year.

The results of the comparison of means for themes of agency and communion in the narratives about turning points, life challenges, failures (regrets), and major events from the past year are graphically presented in Figures 1, 2.

The last analyses explored the differences between individuals with low vs. high levels of compassion in terms of the grammatical forms they used. A series of *t*-tests revealed no statistically significant differences in the frequency of singular and plural verbs, as well as pronouns, in the first, second, and third person. Therefore, in the analyzed linguistic indicators, we did not find any between-group differences in the formal properties of language.

## 4 Discussion

The findings of this study reveal notable insights into how individuals with varying levels of compassion construct narratives about significant life events. Our analysis focused on several narrative characteristics, including affective tone, the frequency of agency and communion themes, and the use of redemption or contamination sequences. We hypothesized that individuals with higher compassion, more than those with lower compassion, would emphasize communion and agency themes in their narratives, that their narratives about difficult life events would have a more positive affective tone, and would more frequently use redemption sequences. Additionally, we expected that individuals with higher compassion would use first-person and second-person plural pronouns and verbs more frequently in their narratives. Our hypotheses were partially confirmed, suggesting that the degree of compassion a person possesses is reflected in how they understand and describe their experiences in self-narratives.

The differences in the themes of communion and agency between individuals with low and high levels of compassion were consistent with our expectations. Study participants with higher levels of compassion more frequently described their narratives about life events related to failure and the past year's most difficult event with themes of love, friendship, and care toward others involved in their stories, reflecting a higher presence of communion. These narratives often illustrated strong connections and a sense of community, emphasizing the importance of relationships and mutual support. Individuals with higher compassion also depicted themselves as proactive agents, focusing on problem-solving and action-oriented behaviors. They framed their experiences around personal efficacy, control, and overcoming challenges. This indicates a strong presence of agency in their narratives, where they saw themselves as active participants in shaping their lives, making decisions, and driving outcomes. These findings together seem consistent with Gilbert's concept of compassion (Gilbert, 2017a; Gilbert et al., 2017), which involves two primary processes: compassionate engagement and compassionate action. Highly compassionate individuals are characterized by both an orientation toward others and a sensitivity to their suffering, as well as the ability and motivation to actively engage in efforts to alleviate or prevent suffering. These two aspects of compassion, as reflected in our results, are mirrored in the way highly compassionate individuals describe and narrate their challenging life experiences, clearly highlighting themes of communion and agency.

It is worth noting that the differences presented above were significant only for two out of the four types of life stories the participants shared: failure and the past year's most difficult event. For narratives about turning points and life challenges, the differences were not statistically significant. This discrepancy may be due to the relatively small sample size, where the slightly larger intra-group variance in the degree of agency and communion themes used in narratives may have reduced the effect of compassion levels below the significance threshold. Nonetheless, this difference was observed. It might be related to the fact that many participants described less stressful events as turning points or challenges compared to those related to failure and the most difficult events of the past year. This could indicate that compassion is primarily associated with incorporating themes of agency and communion in narratives when they pertain to experiences involving high levels of distress, suffering, and negative emotions—experienced by the narrator or other participants in the story. However, this area requires further research and reflection.

Interestingly, we found no significant differences in the affective tone of narratives between individuals with high and low levels of compassion. This suggests that while compassionate individuals may generally experience higher levels of wellbeing and fewer negative emotions (Asano et al., 2020; Gilbert et al., 2017), this does not necessarily translate into a more positive affective tone in their narratives about specific stressful events. This finding aligns with the idea that high compassion may involve a complex emotional experience, including mixed feelings such as empathy and sadness when witnessing others' suffering, or fear and gratitude when they themselves experience stressful events. It can be expected that compassionate individuals will not generally feel more positive emotions when witnessing someone else's suffering or dealing with their own difficult and stressful situations, even if they more frequently experience feelings like sympathy, concern, or tenderness compared to individuals with lower levels of compassion.

The emotional responses of more compassionate witnesses to another person's suffering are likely to be more complex and contextually appropriate. According to Hoffman's (2000) theory, individuals with higher levels of moral development are more likely to experience reactive empathy as opposed to analogous empathy. Their responses to others' suffering involve the observer's emotional reaction to the situation rather than mirroring the exact emotions of the person in distress. Such a reaction includes not only sadness at seeing a crying person but also concern, compassion, and the courage to take actions to help that person. Therefore, the emotions experienced may be mixed rather than unequivocally negative or positive. Future research could aim to better understand the relationships between moral development, compassion, and reactive empathy, and how these relate to compassion competencies such as sensitivity to distress, empathy, helpful attention, imagery, and reasoning.

The study results also show that narratives of individuals with high and low levels of compassion do not significantly differ in terms of formal aspects such as the frequency of specific pronouns and verb types. Our findings suggest that while the formal and linguistic aspects of narratives do not differ significantly between individuals with varying levels of compassion, the content and significance of specific motifs and themes do vary. Future research should delve deeper into this area of analysis and compare the narratives of individuals differing in compassion concerning other indicators, such as coherence, which may be associated with greater cognitive complexity.

### 4.1 Limitations and future research directions

The mixed-method approach used in this study, combining quantitative questionnaire measures with qualitative narrative analysis, offers significant advantages but also has important limitations, which should be considered alongside the methodological strengths of the study.

As is typical for qualitative methods, narrative analysis is inherently subjective and relies on the interpretative skills of the researchers, which may introduce bias. It is worth noting that our interrater reliability analysis indicated acceptable, though not perfect, agreement among coders.

The Life Story Interview procedure used in this study is a widely adopted method, and the approaches to coding narrative properties, such as affective tone and the motivational themes of agency and communion, are well-established in the literature (Adler et al., 2017). However, adhering to these standard practices limited our ability to probe further into participants' accounts. Participants were asked to narrate their stories as they perceived them, without interviewer intervention to clarify ambiguous points or explore how the protagonist interpreted specific aspects of their experiences.

According to Gilbert et al. (2017), compassion comprises three distinct flows: showing compassion to others, receiving compassion from others, and self-compassion. While these aspects are interrelated, they are not identical. Future research should not only explore how low vs. high levels of overall compassion are expressed in narrative properties but also investigate these relationships separately for each flow of compassion. In this study, we adopted a simplified approach by comparing two groups of individuals with relatively low and high levels of compassion, measured as the mean of all three flows. This allowed for the practical grouping of participants but did not account for individual differences in the levels of each flow. Our analysis showed that participants in the “low compassion” and “high compassion” groups differed significantly not only in their average compassion scores but also in the levels of each of the three flows (*p* < 0.01). Nevertheless, future studies could provide deeper insights into how specific narrative properties are associated with each flow of compassion.

A similar issue arises when considering potential differences between the two processes of compassion proposed by Gilbert: motivated attention to suffering (compassionate engagement) and motivated efforts to alleviate and prevent suffering (compassionate action). For instance, it could be hypothesized that at least one competency of compassionate action—behaviors—might be reflected in a greater tendency to construct narratives about difficult events centered on the motivational theme of agency. Conversely, the theme of communion might characterize the narratives of individuals engaging in competencies such as motivation to care and empathy (compassionate engagement) or helpful attention (compassionate action). Analyzing such associations would require a more complex methodology in future studies.

This study's limitations include the sample size, which may have limited the ability to detect medium effect sizes. An a priori power analysis for an independent sample *t*-test, with α = 0.05, power = 0.80, and a one-sided test, determined that for two research groups both about 30 participants, the required effect size (Cohen's *d*) is ~0.72. Given the sample sizes in our study, there was therefore a relatively low probability of detecting a medium effect size if it exists.

Future research should conduct qualitative analyses to identify themes that are more typical for individuals with varying levels of the three flows of compassion, enabling a better understanding of their relationships and the meanings attributed to experiences described in self-narratives. Moving beyond narrative methodologies, future qualitative studies employing in-depth interviews could provide data for more detailed analyses of how the themes of agency and communion are understood depending on the levels of the different flows of compassion, as well as the processes of compassionate engagement and compassionate action. These analyses could also explore related competencies, such as motivation to care, sensitivity to distress, empathy, compassion, distress tolerance, non-judgment, helpful attention, imagery, reasoning, sensory focus, feelings and emotions, and behaviors.

It is also worth exploring the coherence of narratives among individuals with different levels of compassion. Additionally, examining how compassion training influences narrative identity could provide deeper insights into the interplay between compassion and narrative identity.

### 4.2 Implications

Our findings provide new insights, indicating relationships between compassionate engagement and compassionate actions and the tendency to frame narratives about stressful life events around themes of agency and communion. The results imply that individual differences in compassion may influence or manifest in distinctive ways of understanding one's own and others' life experiences. Individuals with high compassion construct narrative representations of experiences differently from those with low compassion. Narrative is a form of understanding oneself and the world (Gergen and Gergen, 1986; Schiff, 2017), and the level of compassion appears to influence the elements upon which this self-understanding is built. The narrative identity of a highly compassionate person includes more content related to seeing oneself as sensitive to suffering and actively working to reduce or prevent it. These results suggest that highlighting themes of agency and communion in self-narratives may serve as an indicator of differences in compassion levels. This can be valuable in designing scientific research in this area, using narrative and qualitative approaches.

The results have significant implications for practitioners of Compassion-Focused Therapy (Gilbert, 2009; Gilbert and Simos, 2022) and those supporting the development of compassion. Developing compassion can enhance various techniques for fostering compassionate engagement and compassionate action, including imaginative techniques. Gilbert and Choden (2013) note that within every individual, there are various potential patterns that serve as sources for different life scenarios, and we can be responsible for shaping these patterns. This can be achieved through engagement with culturally present narratives that offer guidance on what constitutes a good life (McLean et al., 2019). Many inspiring stories depict individuals who are not only sensitive to others' suffering but also take courageous steps to change themselves and the world for the better. Recognizing themes of communion and agency in these narratives can help leverage their impact more effectively. Moreover, knowledge about the importance of individuals' sense of agency and communion can also be used in client work or personal development through narrative techniques, such as writing about experiences in narrative form or sharing these experiences with others. Emphasizing the presence of communion and agency motifs in these self-narratives can promote the development of competencies characteristic of compassionate individuals.

### 4.3 Conclusion

Overall, this study contributes to our understanding of how compassion relates to representing experience in narrative form. Individuals with higher levels of compassion showed more frequent themes of agency and communion. These findings underscore the importance of narrative analysis in understanding the psychological constructs of compassion. Additionally, the study highlights how narrative approaches can be used to foster and support developing compassionate engagements and actions, potentially leading to improved therapeutic practices and personal development strategies. By incorporating narrative techniques, individuals and practitioners can enhance the understanding and cultivation of compassion, ultimately contributing to greater emotional and psychological wellbeing.

## Data Availability

The raw data supporting the conclusions of this article will be made available by the authors, without undue reservation.
